# The matrix metalloproteinase ADAM10 supports hepatitis C virus entry and cell-to-cell spread via its sheddase activity

**DOI:** 10.1371/journal.ppat.1011759

**Published:** 2023-11-15

**Authors:** Belén Carriquí-Madroñal, Julie Sheldon, Mara Duven, Cora Stegmann, Karsten Cirksena, Emanuel Wyler, Francisco J. Zapatero-Belinchón, Florian W. R. Vondran, Gisa Gerold

**Affiliations:** 1 Department of Biochemistry & Research Center for Emerging Infections and Zoonoses (RIZ), University of Veterinary Medicine Hannover, Hanover, Germany; 2 Institute for Experimental Virology, TWINCORE, Centre for Experimental and Clinical Infection Research, a joint venture between the Medical School Hannover and the Helmholtz Centre for Infection Research, Hanover, Germany; 3 Max Delbrück Center for Molecular Medicine in the Helmholtz Association (MDC), Berlin Institute for Medical Systems Biology (BIMSB), Berlin, Germany; 4 Gladstone Institutes, San Francisco, California, United States of America; 5 Department of General, Visceral and Transplant Surgery, Regenerative Medicine and Experimental Surgery, Hannover Medical School, Hannover, Germany; 6 German Center for Infection Research Partner Site Hannover-Braunschweig Hannover, Germany; 7 Wallenberg Centre for Molecular Medicine (WCMM), Umeå University, Umeå, Sweden; 8 Department of Clinical Microbiology, Virology, Umeå University, Umeå, Sweden; Thomas Jefferson University - Center City Campus: Thomas Jefferson University, UNITED STATES

## Abstract

Hepatitis C virus (HCV) exploits the four entry factors CD81, scavenger receptor class B type I (SR-BI, also known as SCARB1), occludin, and claudin-1 as well as the co-factor epidermal growth factor receptor (EGFR) to infect human hepatocytes. Here, we report that the disintegrin and matrix metalloproteinase 10 (ADAM10) associates with CD81, SR-BI, and EGFR and acts as HCV host factor. Pharmacological inhibition, siRNA-mediated silencing and genetic ablation of ADAM10 reduced HCV infection. ADAM10 was dispensable for HCV replication but supported HCV entry and cell-to-cell spread. Substrates of the ADAM10 sheddase including epidermal growth factor (EGF) and E-cadherin, which activate EGFR family members, rescued HCV infection of ADAM10 knockout cells. ADAM10 did not influence infection with other enveloped RNA viruses such as alphaviruses and a common cold coronavirus. Collectively, our study reveals a critical role for the sheddase ADAM10 as a HCV host factor, contributing to EGFR family member transactivation and as a consequence to HCV uptake.

## Author summary

The epidermal growth factor receptor (EGFR) family members are host factors for several viruses including hepatitis C virus (HCV). In the context of HCV infection, EGFR activation and internalization is critical for HCV entry. A known mechanism of transactivation of EGFR family receptors via EGF or E-cadherin shedding involves the disintegrin and matrix metalloproteinase 10 (ADAM10). In this study, we use pharmacological inhibition, siRNA-mediated silencing, genetic ablation of ADAM10 and complementation with the EGFR family member substrates EGF and E-cadherin to show that ADAM10 supports HCV entry and cell-to-cell spread via EGFR family member transactivation.

## Introduction

Hepatitis C virus (HCV) was identified in 1989 in patients who developed non-A, non-B hepatitis after receiving blood transfusions [1–3]. According to WHO estimates, 58 million people globally suffer from chronic hepatitis caused by HCV, and 1.5 million new infections occur every year. While 30% of acutely infected patients spontaneously clear the infection, the majority of exposed individuals develops chronic hepatitis, with a subfraction suffering from cirrhosis and yet a subfraction progressing to hepatocellular carcinoma [4]. The current treatment is a combination of direct acting antivirals (DAAs) that clear HCV in over 95% of the patients [5]. Nevertheless, resistance-associated substitutions in the HCV genome can emerge [6]. In addition, reinfection after DAA treatment is possible and observed in high risk groups including people who inject drugs. Furthermore, immunological alterations like elevated levels of interferon alpha (IFN-α) and survival of memory HCV-specific CD8+ T cells persist after DAA-mediated HCV clearance [7,8]. While only a preventive vaccine would address these challenges, it is not fully understood, why specific individuals progress to chronic infection and severe liver disease. A better understanding of the host factors contributing to susceptibility to HCV may help developing individualized risk assessment strategies and therapies in the future.

To enter human hepatocytes, the virus uses four critical entry factors: human scavenger receptor class B type I (SR-BI, gene product of *SCARB1*), the tetraspanin CD81 and the two tight junction proteins claudin-1 and occludin [9–14]. The virus can also use the low-density lipoprotein receptor (LDLR) when SR-BI is not present and several other entry co-factors facilitate the uptake process of HCV [15]. The latter include the CD81 interaction partners epidermal growth factor receptor (EGFR), transferrin receptor (TfR), E-cadherin, calpain 5 (CAPN5), casitas B cell lymphoma-b (CBLB) and the serum response factor binding protein 1 (SRFBP1) [16–20]. In particular, the role of EGFR is strengthened by the fact that EGFR inhibitors strongly reduce viral load in small animal models of hepatitis C. At a molecular level, the activation of EGFR drives lateral translocation of the virus-CD81 complex towards tight junctions, where the virus interacts with claudin-1 and presumably occludin. This mechanism involves HRas activation downstream of EGFR. Specifically, EGFR signal transduction through HRas and extracellular signal-regulated kinase (ERK) induces the surfing of the virus-receptor complex on the membrane of polarized hepatocytes and promotes HCV uptake at tight junctions of 3D organoids [21–23]. Several entry factors including claudin-1, occludin and EGFR play a role not only in cell-free HCV entry but also in cell-to-cell spread, a mechanism by which HCV can directly infect neighboring hepatocytes, thereby evading neutralizing antibodies [24,25]. Importantly, how EGFR activation is regulated during HCV entry and spread remains largely elusive.

EGFR family member activation in uninfected cells is regulated—among other mechanisms—by plasma membrane lipids and by ‘sheddases’, which release the receptor substrates epidermal growth factor (EGF) and E-cadherin from its membrane-anchor to the extracellular milieu [26,27]. The ‘a disintegrin and metalloproteinase’ (ADAM) sheddase family consists of 21 transmembrane proteins that proteolytically process ectodomains of membrane-bound proteins and bind to integrins or components of the extracellular matrix through their disintegrin domain [28]. In humans, not all the members of the family display proteolytic activity, but ADAM10 and ADAM17 constitute typical examples of ADAMs with sheddase function [29,30]. Among the substrates for ADAM10 and ADAM17 are EGFR family member ligands such as EGF and E-cadherin [31,32]. While ADAM17 function is critical for the assembly of the human papillomavirus (HPV) entry platform, both ADAM10 and ADAM17 are SARS-CoV-2 entry co-factors [33,34].

Here, we identify the EGFR family member activating sheddase ADAM10 as a CD81 interaction partner and HCV host factor. Our data show that ADAM10 pharmacological inhibition, siRNA mediated silencing, and CRISPR/Cas9 mediated gene editing decrease HCV infection. Specifically, ADAM10 inhibition reduces HCV entry and cell-to-cell spread. Mechanistically, we show that the soluble ADAM10 substrates EGF and E-cadherin rescue HCV infection in *ADAM10* knockout cells. Taken together, our data suggest that ADAM10-mediated EGF and E-cadherin shedding is required to activate EGFR family members and consequently promote susceptibility to HCV.

## Material and methods

### Ethics statement

Culturing and experimentation using primary human hepatocytes was approved by the ethics commission of the Hannover Medical School (vote # 3319–2016). Patients were adults and gave written informed consent. This study was performed according to the ethical standards approved in the 1975 Declaration of Helsinki.

### Cell culture

The cell line Huh-7.5 expressing Firefly luciferase (Huh-7.5-Fluc) was cultured in Dulbecco´s modified Eagle´s medium supplemented with 10% fetal calf serum (Capricorn scientific), 1% penicillin/streptomycin (Gibco), 1% L-glutamine (Gibco), 1% non-essential amino acids (Gibco) and blasticidin (Fisher Scientific, 5 μg/mL) [35]. Huh-7.5-Fluc cells were cultured at 37°C and 5% CO_2_. To detect intracellular ERK1/2 phosphorylation levels, Huh-7.5-Fluc cells were cultured in RPMI 1640 medium (Gibco) without supplements and stimulated with soluble EGF (Sigma). Primary human hepatocytes (PHH) were isolated from explanted livers as previously reported [36], plated on collagen-coated 6 well plates at a density of 1x10^6^ cells/well and cultured in hepatocyte culture medium HCM (Lonza). Protocols were approved by the ethics commission of Hannover Medical School, Hannover. Written informed formal consent was given by patients.

### Cell-to-cell spread assay

To assess HCV cell-to-cell spread, Huh-7.5-Fluc cells were infected with GT2a GFP-tagged HCV Jc1/NS5A/B-EGFP (MOI = 0.1) for one week [37]. In parallel, Huh-7.5-Fluc cells were pretreated with GI254023X, BB-94 or DMSO for 16 h prior to mixing with infected cells at a 1:5 ratio of infected to uninfected cells. 4 h after mixing, cells were overlayed with 2.4% Avicel (CL-611 NF, MCC/carboxymethylcellulose sodium) (IMCD) diluted in H_2_O. The overlay was supplemented with GI254023X, BB-94 or dimethylsulfoxid (DMSO). HCV cell-to-cell spread was quantified by flow cytometry as the percentage of GFP-positive cells. Data were then normalized to the percentage of GFP-positive cells in the DMSO control condition.

### Cell viability assay

To assess cell viability, cells were treated with 5 mg/mL of 3-(4,5-Dimethylthiazol-2-yl)-2,5-Diphenyltetrazoliumbromid (MTT) (Thermo Fisher Scientific) diluted in DMEM medium (1:100) for 2h. Medium was then replaced with 50 μL of DMSO and absorbance was measured at 570 nm using a spectrophotometer (Multiskan Go, Thermo Fisher Scientific).

### Inhibitor preparation

The ADAM10 inhibitor GI254023X (Merck), the JAK/STAT inhibitor Ruxolitinib (AdipoGen Life Sciences), the broad-spectrum matrix metalloproteinase inhibitor Batimastat (BB-94) (Calbiochem), the SR-BI inhibitor ITX5061 (Sigma) and the HCV protease inhibitor telaprevir (Sigma) were diluted in DMSO (99.5%, Roth). Ruxolitinib, BB-94 and ITX5061 were diluted to a stock concentration of 10 mM. GI254023X was diluted to a stock concentration of 2.6 mM.

### Pseudoparticle transduction and infection assays

HEK293T cells were cotransfected at an equimolar ratio of pCMVΔR8-74 (Addgene #22036), pWPI vector expressing a firefly luciferase reporter and phCMVcE1E2 (H77) (GT1a) or pc.Z.VSVG to produce lentiviral pseudoparticles (termed HCVpp or VSVpp, respectively) as described elsewhere [20] [38–41]. Huh-7.5 cells were transduced with lentiviral pseudoparticles for 72 h and transduction efficiency was measured by firefly luciferase assay using a plate reader (Centro XS^3^ LB 960, Berthold) as described previously [42].

Infectious cell culture-derived HCV (HCVcc) was produced by electroporation of Huh-7.5.1 [43] cells with *in vitro*-transcribed RNA (gt-2a/2a chimera (Jc1) encoding Renilla luciferase (JcR2a) or GT2a Jc1/NS5A/B-EGFP), following a previously published protocol [35] [37,42,44]. In general, Huh-7.5-Fluc cells were infected with HCVcc for 72 h. To assess infectivity, Renilla luciferase activity was measured using coelenterazine as substrate. In parallel, Firefly luciferase values were measured to assess cell counts. Infection values were then normalized to cell count values. Luciferase assays were performed as described previously using a plate reader (Centro XS^3^ LB 960, Berthold) [42].

The cell culture adapted HCV, p100pop was produced as previously described [45]. New stocks were generated by infecting Huh-7.5.1 cells and collecting 45 μM filtered viral supernatant 60–80 hours post infection (hpi). PHH were treated with 10 μM GI254023X with or without Ruxolitinib (10 μM) for 24 h and subsequently infected with 3.75x10^5^ TCID50 of P100pop (measured on Huh-7.5 cells). Four hpi, cells were washed five times with PBS and HCM media containing the inhibitors or DMSO was added. Supernatant and cells lysates were collected 4, 24 and 72 hpi for infectious particle release quantification using TCID50 as previously described [46].

The recombinant human coronavirus hCoV-229E encoding a Renilla luciferase was used for infection of Huh-7.5-Fluc cells, as well as the alphaviruses VEEV (TC-83 strain, MOI 0.1) and CHIKV (LR2006-OPY1 ECSA genotype, MOI 1) encoding EGFP under a subgenomic promoter [47–52], which were kindly provided by Ilya Frolov and Graham Simmons. Huh-7.5-Fluc cells were seeded at a density of 1.5x10^4^ cells/well in a 96 well plate format. 24 h later, cells were treated with 1, 5 or 10 μM ADAM10 inhibitor and infected 24 h later in continued presence of ADAM10 inhibitor. Infectivity was measured 48 h post infection (hCoV-229E) by luciferase measurement or continuously during the first 24 h of infection (VEEV and CHIKV) using the IncuCyte S3 imaging platform (Sartorius) to measure GFP expression.

### Rescue with soluble ligands

During rescue experiments, Huh-7.5-Fluc ADAM10 knockout or control cells lines were treated with 50 ng/mL tumor necrosis factor (TNF-α, BioLegend), 1 μg/mL EGF (Sigma) or 8 μg/mL E-cadherin (R&D Systems) for 16 h prior to and during infection with HCVcc. 72 h post infection, infectivity was measured performing Renilla luciferase assay as described above [42]].

### HCV replication assays

Huh-7.5-Fluc cells were electroporated with *in vitro* transcribed RNA generated from an HCV subgenomic replicon expressing the non-structural proteins of HCV GT 1b isolate Con1 together with firefly luciferase and a replication-deficient variant of the same replicon (ΔGDD) [35] following a previously published protocol [42].

### Flow cytometry

Huh-7.5-Fluc cells or PHH were fixed using 1.5% paraformaldehyde (PFA) diluted in PBS supplemented with 1% FCS. For cell surface expression detection, Huh-7.5-Fluc cells were stained with APC-conjugated mouse monoclonal antibodies against ADAM10 (Biolegend, clone SHM14), CD81 (BD Biosciences, clone JS-81), SR-BI (Biolegend, clone m1B9) and ADAM17 (Cell Signaling Technology). For measurement of intracellular protein levels, cells were permeabilized using PBS supplemented with 1% FCS and 0.1% saponin followed by staining against occludin (Thermo Fisher Scientific clone OC-3F10) or claudin-1 (Thermo Fisher Scientific, rabbit polyclonal). Secondary antibodies used were anti-mouse or anti-rabbit AF647 (Thermo Fisher Scientific). For measurement of intracellular ERK1/2 and EGFR phosphorylation levels, cells were permeabilized using ice-cold methanol and stained with APC-conjugated mouse monoclonal antibody against phosphorylated ERK1/2 (pERK1/2) (Biolegend, clone 6B8B69) or with antibody against phosphorylated EGFR (pEGFR) (Cell Signaling Technology), respectively. As isotype controls, APC-conjugated mouse IgG1κ and mouse IgG2aκ were used. Flow cytometric measurements were performed on an Attune NxT acoustic focusing cytometer (Invitrogen, Thermo Fisher Scientific).

### Immunoblot

Huh-7.5-Fluc cells were lysed for 30 min on ice in cell lysis buffer prepared with 1% NP40 (Sigma), 50 mM Tris-HCl, pH 7.4 (Carl Roth), 62.5 mM EDTA (Carl Roth), 0.4% Na-deoxycholate (Carl Roth). Protein content was determined using Bradford assay [53] and 10 μg of protein were boiled for 5 min at 95°C in SDS sample buffer under reducing conditions. As an exception, cell lysates used for CD81 detection were boiled in SDS sample buffer under non-reducing conditions. Samples were resolved by SDS-PAGE and blotted onto a PVDF membrane. Staining with primary antibodies was performed o/n at 4°C using antibodies against CD81 (SantaCruz, clone 1.3.3.22), ADAM10 (Cell Signaling Technology), pERK1/2 (Cell Signaling Technology), ERK1/2 (Cell Signaling Technology), pEGFR (Cell Signaling Technology), vinculin (Merck, clone 3M13) and actin (HRP-conjugated, Sigma, clone AC-15). As secondary antibodies, we used anti-mouse or anti-rabbit IgG HRP-conjugated antibodies (Sigma).

### Small interfering RNA (siRNA)-mediated knockdown experiments

Huh-7.5-Fluc cells were seeded in 24 well plates at a density of 2x10^5^ cells/well and cultured for 24 h before transfection with 7.5 pmol of a pool of three different siRNAs targeting ADAM10 mRNA (Thermo Fisher scientific; siRNA IDs: s1004, s1005, s1006) or ADAM17 mRNA (Thermo Fisher scientific; siRNA IDs: s13719, s13718, s13720) using Lipofectamine RNAiMAX (Thermo Fisher scientific) according to manufacturer´s instructions. Media was replenished 24 h after and cells were cultured for further 24 or 48 h, as indicated.

### CRISPR/Cas9 knockout generation

The pLentiCRISPR v2 ccdB plasmid [54] (Addgene #52961) was used as a vector backbone. The single guide RNA (sgRNA) sequence 5’-CTTCGATGCAAATCAACCAGG-3’ (selected using the software CHOPCHOP [55]) was cloned into the vector backbone under the control of the U6 human promoter and lentiviral particles encoding this sgRNA sequence as well as *Cas9* were generated as previously described [16]. The non-targeting sgRNA sequence 5’-CTAAGGTTAAGTCGCCCTCG-3’ was cloned in parallel as negative control.

### Generation of ADAM10 overexpressing cell line

The ADAM10 coding sequence (isoform 1, NCBI accession number: NM_001110.4) was cloned into the pWPI plasmid (Addgene #12254) using the Gibson assembly cloning strategy [56] according to the manufacturer´s instructions and following a previously described protocol [16]. Lentiviral particles pseudotyped with VSVG and encoding the *ADAM10* gene were generated as described above. In parallel, we generated lentiviral particles carrying the pWPI vector backbone sequence (empty vector control). 4x10^4^ Huh-7.5-Fluc cells/well were seeded in a 24-well plate format. The day after seeding, lentiviral transduction was performed adding 500 μL/well of lentivirus containing cell culture supernatant and incubating overnight at 37°C. The lentiviral supernatant was then replaced with DMEM supplemented as described in the cell culture section.

### Single-cell and bulk RNA-sequencing reanalysis

In order to assay expression of HCV entry factors in the different liver cell types, we downloaded published human liver atlas data from GEO entry GSE124395. Cell types were assigned based on the marker genes in the original paper. For each cell type and each gene in question, we quantified the average expression level and the percentage of cells having at least one detected transcript. *ADAM* family member transcript expression levels in primary hepatocytes and hepatoma cells were retrieved from published datasets [57,58].

### Statistical analysis and image generation

Unless otherwise stated, experiments were performed in three biological replicates with three technical replicates each. Data representation and statistical analysis were performed using GraphPad Prism v9.0. Graphs show the mean of three biological replicates +/- standard deviation (SD). Statistical significance was calculated using t-test (unpaired/paired) or using one-way or two-way analysis of variance (ANOVA) followed by Dunnet´s post-hoc test in case of multiple comparisons. Figure layouts were generated using Adobe Illustrator version 26.5. Schematic illustrations were generated using BioRender (BioRender.com).

## Results

### ADAM10 interacts with the HCV entry factor CD81 in liver cells

**Fig 1:**
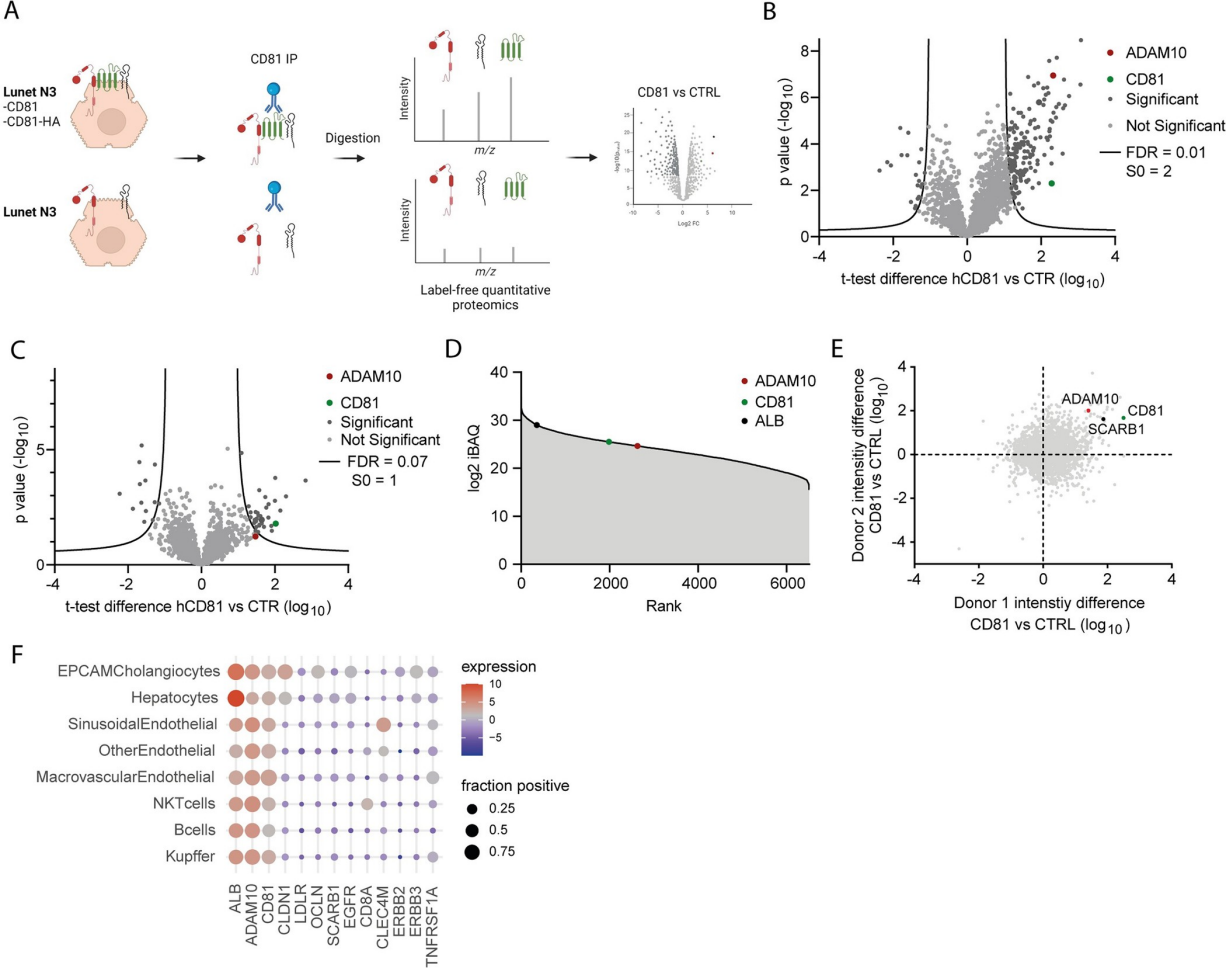
ADAM10 is highly enriched in CD81 affinity purifications from hepatoma cells and primary human hepatocytes. (**A**) Experimental design of affinity enrichment mass spectrometry for the identification of the CD81 protein complex in human hepatoma cell lines (IntAct entry https://www.ebi.ac.uk/intact/search?query=IM-25678). **(B)** Results of two-sample t-tests comparing protein label free quantification (LFQ) intensities of CD81 co-IPS from Lunet N hCD81 cells (CD81) with those of Lunet N (CTRL) displayed as a volcano plot. **(C)** Same as in (B), comparing protein LFQ intensities of HA co-IPS from Lunet N hCD81-HA (CD81) with those of Lunet N (CTRL). **(D)** Ranked protein abundances in whole cell proteomes of human hepatoma cells. **(E)** Protein enrichment in CD81 co-IPs from primary human hepatocytes (PHH) of two independent donors. Isotype control antibodies were used as control (CTRL). **(F)** Transcript expression of *ADAM10*, HCV entry factors, receptors transactivated by ADAM10 (*EGFR*, *ERBB2*, *ERBB3*, *TNFRSF1A*) and negative controls (*CD8A*, *CLEC4M*) across cells in healthy human liver tissue from nine donors using the cell type annotation from Aizarani et al. [57]. The size of the dots represents the percentage of transcript positive cells per cell type with at least one detected transcript for the respective gene, and the colour the average expression in log_2_ scale. Mass spectrometry datasets show median values of four independent experiments for the hepatoma cells. Proteomics data derived from and described in (Bruening et al. 2018) [16].

In a previous study, we analyzed the protein network of the HCV entry factor CD81 by affinity enrichment mass spectrometry (Fig 1A, derived from Bruening et al. (16); IntAct entry https://www.ebi.ac.uk/intact/search?query=IM-25678). We enriched for endogenous CD81 from hepatoma cells or primary human hepatocytes (PHH) with CD81-specific antibodies and performed additional immunoprecipitations with anti-HA antibodies from hepatoma cells ectopically expressing HA-tagged CD81. After label free quantification of enriched proteins, the results were filtered for strong enrichment compared to the respective negative control (30-fold) and false discovery rates (lower than 0.01) in all replicates from PHH and hepatoma cells, respectively. Applying these rather stringent statistical cut offs, we found 33 proteins potentially forming a complex with CD81 and, among these, we identified CAPN5 and CBLB as new host factors for HCV. Notably, another highly enriched protein (Fig 1B and 1D), ADAM10 was slightly below the chosen threshold (FDR = 0.07, s0 = 1) in immunoprecipitations from CD81-HA expressing cells (Fig 1C) used in our previous work (16); however, we observed protein enrichment of ADAM10 similar to that of the bait CD81 and the positive control SCARB1 in CD81 pullouts from CD81 expressing cell lines and primary human hepatocytes from two independent donors (Fig 1E). In addition, we show that ADAM10 is broadly expressed in different liver cell types (Fig 1F), and also in hepatocytes and Kupffer cells. This prompted us to investigate a putative role for ADAM10 in HCV infection.

### ADAM10 surface expression is required for full HCV infectivity

**Fig 2:**
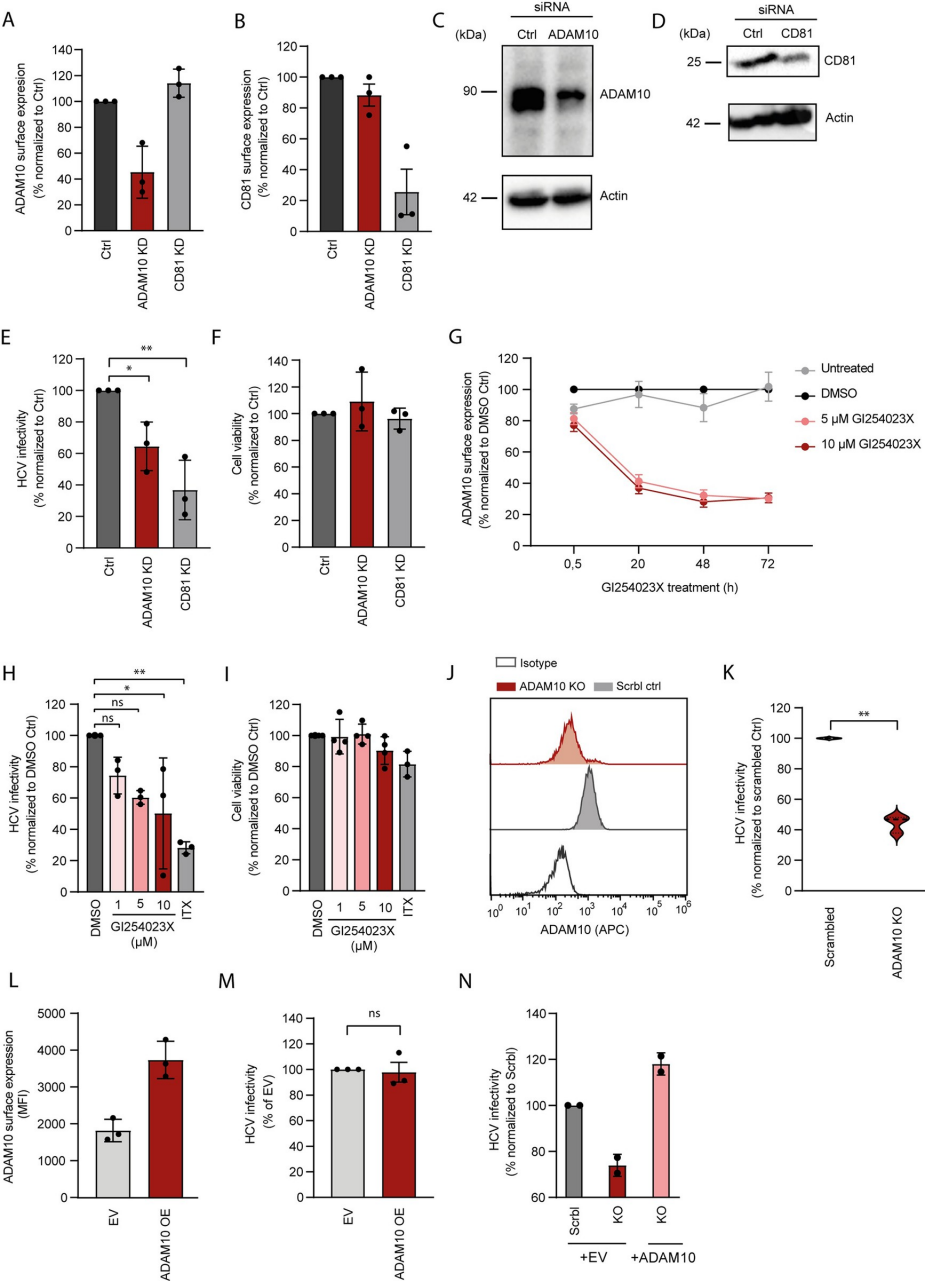
ADAM10 is a host factor for hepatitis C virus. **(A,B)** ADAM10 (A) and CD81 (B) surface expression levels measured by antibody staining and flow cytometry on Huh-7.5-Fluc cells treated with a pool of 3 siRNAs targeting *ADAM10*, *CD81*, or a non-targeting siRNA control. Mean fluorescence intensity (MFI) values normalized to control siRNA are shown. **(C,D)** ADAM10 (C) and CD81 (D) protein expression levels determined by immunoblot of lysates from Huh7.5-Fluc cells treated as in (A). **(E)** Huh-7.5-FLuc cells were treated as in (A) and (B) for 48 h and infected with a Jc1-based renilla luciferase reporter virus (JcR2A). Infectivity was quantified 72 hpi as renilla luciferase activity. Infectivity values are normalized to non-targeting siRNA control. **(F)** Cell viability of cells treated as in (E) was assessed by MTT assay. **(G)**. ADAM10 surface expression in cells treated with the ADAM10 inhibitor GI254023X or DMSO was measured as in (A). **(H)** HCV infection of cells pre-treated with GI254023X or SR-BI inhibitor ITX5061 for 16h. Virus inoculum was supplemented with inhibitors, removed 4 hpi and replaced with medium supplemented with inhibitors. Infectivity was measured and values were normalized as in (E). **(I)** Cell viability upon inhibitor treatment as in (H) was assessed by MTT assay and values were normalized to DMSO control. **(J)** Huh-7.5-FLuc cells were treated with a sgRNA targeting *ADAM10* exon 11 and a non-targeting sgRNA control. ADAM10 expression was measured as in (A). **(K)** ADAM10 KO and control cells were infected as in (E). Infectivity was measured as described in E and values were normalized to non-targeting control. **(L)** Huh-7.5-Fluc cells were transduced with lentiviruses encoding ADAM10 (ADAM10 OE) or control lentiviruses (EV). Surface expression levels of ADAM10 overexpressing cells were quantified by antibody staining and flow cytometry. **(M)** Huh-7.5-Fluc cells were transduced as in (L). 72 h post transduction, cells were infected with JcR2a. 72 h post infection, infection was quantified as in (E). **(N)** ADAM10 KO and control cells were transduced as in (L). Cells were then infected with JcR2a 72 h post transduction. Infectivity was measured 72 hpi as in (E). Data show the mean +/- SD of three biological replicates. One-way ANOVA with Dunnett´s multiple comparison test (E and H). Paired t-test (K and M). * P < 0.05; ** P < 0.01; *** P ≤ 0.001.

The interaction of CD81 with ADAM10 and the evidence showing that ADAM10 regulates activation and shedding of the HCV entry co-factors EGFR and E-cadherin, respectively prompted us to determine if ADAM10 plays a role in HCV infection [17,18,59]. To that end, we silenced ADAM10 expression using a pool of three siRNAs targeting *ADAM10* in Huh-7.5-Fluc hepatoma cells. In parallel, we silenced *CD81* expression or treated cells with non-targeting siRNA as positive and negative controls, respectively. We then used antibody staining and flow cytometry to quantify ADAM10 and CD81 surface expression after siRNA treatment (Fig 2A and 2B). Silencing caused a 75% and 45% decrease in CD81 and ADAM10 surface expression, respectively and was confirmed by immunoblot (Fig 2C and 2D). Next, we determined cell susceptibility to HCV after siRNA knockdown (Fig 2E). As expected, CD81 silencing decreased susceptibility to genotype 2a luciferase reporter cell culture-derived HCV (HCVcc), by 63%. Notably, ADAM10 silencing decreased HCV susceptibility by 36% without affecting cell viability (Fig 2F), suggesting a possible role of ADAM10 in HCV infection. To confirm this, we selected an orthogonal pharmacological approach to decrease ADAM10 surface expression. We treated Huh-7.5-Fluc cells with the ADAM10 active site inhibitor GI254023X, which binds to the Zn^2+^ ion present in the active site of matrix metalloproteinases from the ADAM family. GI254023X is specific for ADAM10 due to its optimized fit into the S_1_´ pocket of ADAM10 [60]. Treatment with GI254023X decreases ADAM10 surface expression and concomitantly proteolytic activity [61]. After treating Huh-7.5-Fluc cells with 5 or 10 μM of GI254023X, we observed a reduction of ADAM10 surface expression by approximately 70% at 48 h and 72 h post treatment compared to cells treated with solvent control (Fig 2G). In line with the results obtained after siRNA silencing, when treating Huh-7.5-Fluc cells with GI254023X 16 h prior to infection and during the whole course of infection, we detected a dose-dependent decrease in susceptibility to HCV infection (Fig 2H). The highest tested dose of ADAM10 inhibitor (10 μM) reduced HCV infection to 59% of solvent control treated cells. The previously characterized SR-BI inhibitor ITX5061 reduced HCV infection by 78%, however it also reduced cell viability by 20%. Notably, GI254023X did not affect cell viability at the concentrations tested (Figs 2H, 2I and S1), suggesting a role of ADAM10 in the HCV life cycle.

### CRISPR/Cas9 gene editing confirms ADAM10 as an HCV host factor

To confirm a role for ADAM10 in HCV infection, we generated Huh-7.5-Fluc CRISPR/Cas9 edited cell batches that largely lack expression of ADAM10. In parallel, we generated a control cell line transduced with a non-targeting sgRNA. ADAM10 knockout cells showed a 90% reduction in surface expression of ADAM10 after antibody staining and flow cytometry compared to non-targeting sgRNA transduced cells (Fig 2J). ADAM10 knockout cells showed a 55% decrease in HCV infection (genotype 2a) compared to the non-targeting control, confirming ADAM10 as an HCV host factor (Fig 2K). In addition, we used lentiviruses encoding ADAM10 to generate an ADAM10 overexpressing Huh-7.5-Fluc cell line (Fig 2L) and lentiviruses that carry an empty vector to produce a control cell line. The ADAM10 overexpressing cells displayed twice as much ADAM10 on the surface as the control cell line. However, HCV infection in the ADAM10 overexpressing cell line was not higher than in the control cell line (Fig 2M). Furthermore, ectopic expression of ADAM10 in ADAM10 KO cells rescues the phenotype on HCV infection (Fig 2N). Collectively, this suggests that ADAM10 levels in hepatoma cells are not limiting HCV infection.

### ADAM10 plays a role in entry and cell-to-cell spread of cell culture-derived HCV

**Fig 3:**
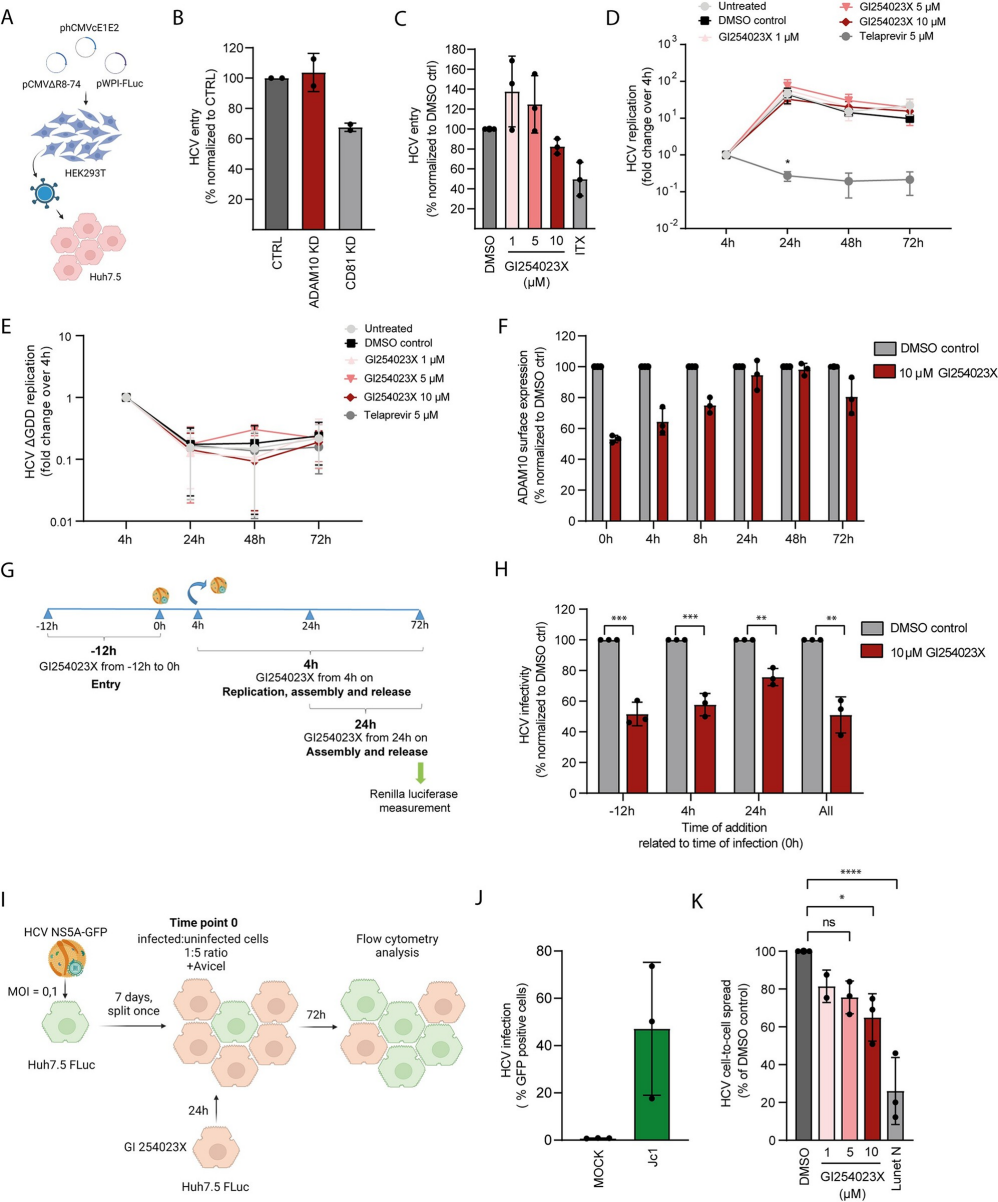
ADAM10 promotes HCV lipoviroparticle entry and cell-to-cell spread but is dispensable for HCV replication and HCV pseudoparticle entry. **(A)** Scheme of the generation of E1/E2 pseudotyped lentivirus. Pseudoparticles encode a firefly luciferase for quantification of transduction efficiency. **(B)** Huh-7.5 cells treated for 48 h with pools of 3 siRNAs targeting *ADAM10* or *CD81* or a non-targeting control (CTRL) were transduced with lentiviruses pseudotyped with HCV E1/E2 glycoproteins. 72 h post transduction, transduction efficiency was measured as firefly luciferase activity. Values represented relative to non-targeting control (CTRL). **(C)** Huh-7.5 cells were treated with the ADAM10 inhibitor GI254023X and the SR-BI inhibitor ITX 5061 for 16 h and transduced with the lentiviruses described in (A). Infectivity was measured 72 h post transduction as in (B). **(D,E)** Huh-7.5-Fluc cells were electroporated with a wildtype (D) or replication deficient ΔGDD mutant (E) HCV subgenomic replicon (SGR) encoding firefly luciferase and treated with ADAM10 inhibitor GI254023X or the NS3/4A inhibitor telaprevir during and after electroporation. HCV subgenome replication was assessed as firefly luciferase activity. Data show the fold change over 4 h luciferase activity values **(F)** ADAM10 surface expression was measured by antibody staining and flow cytometry at the indicated time points post 12 h inhibitor treatment and washout. Mean fluorescence intensity (MFI) values are normalized to DMSO at each time point. **(G)** Scheme of the ADAM10 inhibitor time of addition assay. Huh-7.5-Fluc cells were treated with ADAM10 inhibitor GI254023X or DMSO prior, during or after HCV virus inoculation. **(H)** HCV infection of cells treated with inhibitor as shown in (G). Infectivity was measured by renilla luciferase assay at 72 hpi. Infectivity values are normalized to DMSO controls for each time point. **(I)** Scheme showing the HCV cell-to-cell spread assay. Huh-7.5-Fluc cells were infected with GFP-tagged HCV (MOI 0.1). 7 dpi, Huh-7.5-Fluc GFP+ cells were mixed with uninfected Huh-7.5-Fluc cells pretreated with the ADAM10 inhibitor GI254023X, the matrix metalloprotease inhibitor BB-94, or DMSO at a 1:5 ratio. 4 h later, cells were overlayed with Avicel-containing medium supplemented with inhibitors or DMSO. Infectivity was measured 72 hpi using flow cytometry. **(J)** Percentage of GFP positive cells 7 dpi. **(K)** Cell-to-cell spread of HCV measured as percentage of GFP positive cells in the conditions described in (I) was normalized to DMSO controls. Data shows the mean +/- SD of three biological replicates. Two- way ANOVA with Dunnett´s multiple comparison test (D). Unpaired t-test (H). One-way ANOVA with Dunnett´s multiple comparison test (K). * P < 0.05; ** P < 0.01; *** P < 0.001; **** P < 0.0001.

Our findings showing that ADAM10 is a CD81 interaction partner suggest a possible role of ADAM10 in HCV entry. To study ADAM10 function during early HCV entry steps, we generated lentiviral pseudoparticles expressing the HCV surface glycoproteins E1/E2 (Fig 3A). We treated Huh-7.5 cells with siRNA targeting ADAM10 (Fig 3B) and in parallel with GI254023X (Fig 3C) prior to transduction with lentiviruses expressing HCV E1/E2. Neither GI254023X treatment nor transfection with siRNA targeting ADAM10 impaired pseudoparticle entry. As expected, cells treated with siRNA targeting CD81 or with the SR-BI inhibitor ITX5061 showed decreased pseudoparticle entry. These results indicate that ADAM10 does not play a role in glycoprotein-dependent HCV entry of pseudoparticles, which lack the lipo-viro particle nature of cell culture and patient derived HCV.

Next, we assessed if ADAM10 could impact replication of HCV genomes. To bypass the requirement for virus entry, we electroporated Huh-7.5-Fluc cells with HCV subgenomic RNA and treated the cells with different concentrations of GI254023X. (Fig 3D). Treatment with GI254023X did not decrease HCV replication at any of the concentrations or time points tested. In contrast, treatment with the NS3/4A protease inhibitor Telaprevir significantly decreased HCV replication. Moreover, the replication deficient HCV subgenome (ΔGDD) did not replicate in Huh-7.5-Fluc cells in presence or absence of GI254023X as expected (Fig 3E).

For HCV entry, the association of HCV with serum apolipoproteins in lipo-viro particles is critical for productive uptake [62]. This unique feature of patient derived HCV is not mimicked by HCV pseudoparticles, but only by cell culture derived HCV (HCVcc) and can affect host factor usage and endocytic uptake. Moreover, HCVpp rely on the lentiviral uncoating machinery. Hence, upstream mechanisms that target HCVcc to intracellular compartments allowing uncoating and productive infection are not mimicked by HCVpp [16,20,63]. Consequently, we tested a possible effect of ADAM10 on HCVcc entry using a time-of-addition experiment (Fig 3G). Moreover, the addition of GI254023X at different time points during infection allowed us to clarify if additional steps in the HCV life cycle are affected by ADAM10 depletion (Fig 3G). We first evaluated the kinetics of inhibitor-induced ADAM10 cell surface reduction by treating Huh-7.5-Fluc cells with 10 μM GI254023X and removing the drug 12 h after (Fig 3F). Treatment with GI254023X for 12 h caused a marked decrease in ADAM10 surface expression. The surface levels of ADAM10 started to progressively increase 4 h after inhibitor washout and were comparable to expression levels in the control condition 24 h after GI254023X removal (Fig 3F). As ADAM10 surface levels were still decreased 4 h after inhibitor washout, we proceeded with the time-of-addition experiment as described in Fig 3G. Pretreating the cells with inhibitor for 12 h to target HCVcc entry showed a 50% decrease in HCV infectivity (Fig 3H). In addition, including the inhibitor at later time points targeting replication, assembly or spread impaired HCV infectivity to a similar extent. Adding the inhibitor both at 4 hpi and 24 hpi reduced HCV infection by 40% and 25%, respectively. To exclude off-target effects of the ADAM10 inhibitor, we additionally tested lower doses of the drug. 100 nM and 500 nM refer to 20 or 100 times the IC50 and also caused a dose-dependent reduction of HCV infection (S2 Fig). Together with the subgenomic replicon experiment, these results suggest that ADAM10 plays a role in HCV entry and at a later life cycle step, after replication.

To clarify which late life cycle step is affected by ADAM10 depletion, we focused on cell-to-cell spread, as this step is mechanistically similar to entry. For this purpose, we infected Huh-7.5-Fluc cells with a GFP reporter virus for seven days (Fig 3I) and mixed infected cells with non-infected cells (treated with GI254023X) at a 1:5 ratio before adding an overlay to prevent cell-free HCV spread (Fig 3I). On average, 47% of the cells were infected seven days post infection (dpi) (Fig 3J). Of note, the variability in number of HCV positive donor cells between biological replicates did not influence the outcome of the subsequent cell-to-cell spread assay. Treatment with GI254023X caused a dose-dependent decrease in HCV cell-to-cell spread, confirming a role of ADAM10 in cell-to-cell spread (Fig 3K). We included Lunet N cells (lacking endogenous CD81 expression) as a negative control. As expected, we did not observe HCV cell-to-cell spread into Lunet N cells (Fig 3K). In sum, our data indicate that ADAM10 affects HCV cell entry and spread.

### Broad-spectrum matrix metalloproteinase inhibitors reduce ADAM10 surface levels and HCV infection

**Fig 4:**
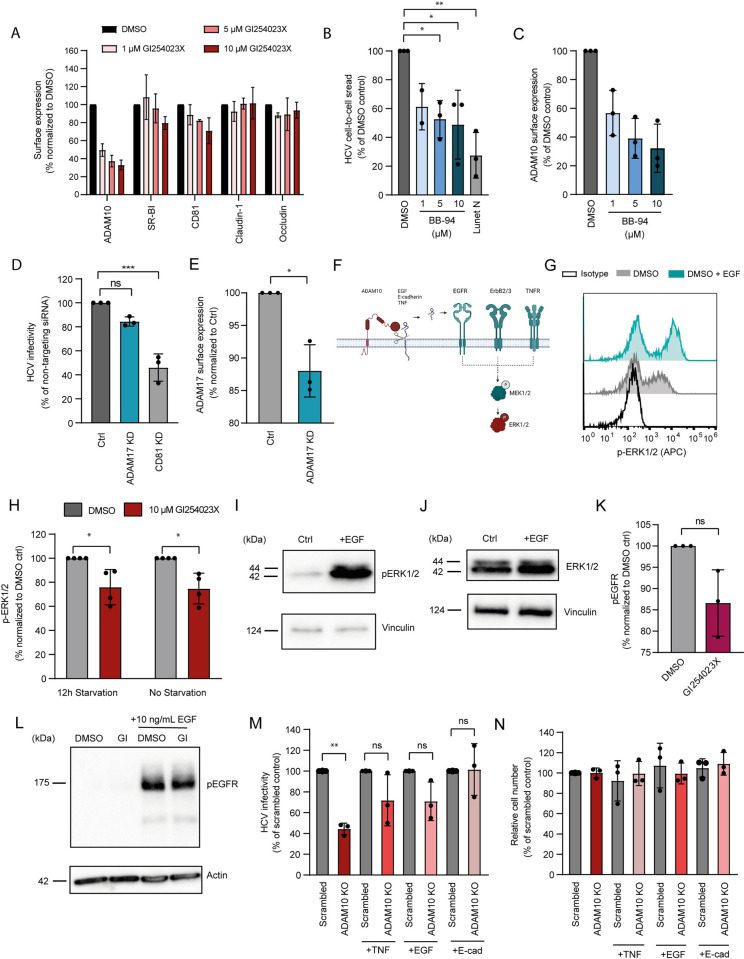
ADAM10 sheddase activity promotes HCV infection. **(A)** Surface expression of HCV entry factors on Huh-7.5-Fluc cells treated with the ADAM10 inhibitor GI254023X or DMSO for 72 h. Values represent mean and SD of the mean fluorescence intensity of three independent experiments and are presented as percentage of DMSO control values. **(B)** Cell-to-cell spread of HCV in Huh-7.5-Fluc cells treated with the matrix metalloprotease inhibitor BB-94 was quantified as in Fig 3K. **(C)** ADAM10 surface expression upon BB-94 or DMSO treatment quantified as in (A). **(D)** HCV infection of Huh-7.5-Fluc cells treated with non-targeting siRNA control or pools of 3 siRNAs targeting *ADAM17* or *CD81*. 48 h post siRNA transfection, cells were infected with JcR2a for 72 h. Infection levels were measured by renilla luciferase assay and normalized to the non-targeting control. **(E)** Huh-7.5-Fluc cells were treated as in (D). 48 h post siRNA transfection, ADAM17 surface expression levels were analyzed by antibody staining and flow cytometry **(F)** Scheme of receptors and downstream kinases activated by ADAM10 sheddase activity. **(G)** Representative histogram showing increased ERK1/2 phosphorylation upon EGF stimulation in DMSO-treated, serum-starved cells. **(H)** ERK1/2 phosphorylation in Huh-7.5-Fluc cells treated with ADAM10 inhibitor GI254023X or DMSO for 48 h, serum starved for 12 h or left in medium containing serum and stimulated with 1 μg/mL EGF for 15 min. Values represent mean and SD of the mean fluorescence intensity of four independent experiments and presented as percentage of DMSO control values. **(I,J)** pERK1/2 (I) and total ERK1/2 (J) protein levels analyzed by immunoblot from Huh-7.5-Fluc cell lysates after serum starvation and EGF stimulation for 15 min as in (G). **(K,L)** pEGFR levels measured by flow cytometry (K) or immunoblot (L) in Huh-7.5-Fluc cells treated with ADAM10 inhibitor GI254023X or DMSO for 48 h, serum starved for 12 h and stimulated with EGF for 15 min. Mean fluorescence intensity analysis as in (A). **(M)** Rescue of ADAM10 KO cell infection phenotype by pretreatment 16 h prior to and during HCV infection with the ADAM10 substrates TNF-α, EGF, or E-cadherin. Infection measured as renilla luciferase activity as in (D) and values normalized to the respective scrambled guide RNA control. **(N)** Relative cell number of ADAM10 KO and control cells upon treatment with ADAM10 substrates as in (M). Values normalized to scrambled guide RNA control cells. Data show the mean +/- SD of three biological replicates unless stated otherwise. One-way ANOVA with Dunnett´s multiple comparison test (B, D). Paired t-test (, E, H, M). * P < 0.05; ** P < 0.01; *** P < 0.001.

As HCV entry requires the expression of CD81, SR-B1, CLDN1 and OCLN, we monitored the expression of the four HCV entry factors after treatment with GI254023X. We treated Huh-7.5-Fluc cells with 1, 5 and 10 μM GI254023X for 72 h and measured the expression of the HCV entry factors in parallel to ADAM10 surface expression by antibody staining and flow cytometry (Fig 4A). Although expression of claudin-1 and occludin was not affected by treatment with GI254023X, cell surface expression of SR-B1 and CD81 was diminished after drug treatment. In the case of SR-BI, surface expression remained above 80% of DMSO control and cell surface expression of CD81 was only affected at the highest concentration of GI254023X with a reduction to 71% of controls. In this particular case, variability was high between biological replicates with two of the biological replicates presenting 80% residual surface expression compared to DMSO control. Taking published threshold data into account [64], we conclude that the decrease in susceptibility to HCV after ADAM10 inhibition cannot be explained solely by a decrease in HCV entry factor expression.

To confirm a role for matrix metalloproteinases in HCV infection, we included a broad-spectrum matrix metalloproteinase inhibitor (BB-94) in our cell-to-cell spread assay. Again, we infected Huh-7.5-Fluc cells with a GFP reporter virus and mixed infected cells with non-infected cells (treated with BB-94) at a 1:5 ratio as shown in Fig 3I. Treatment with 5 and 10 μM of BB-94 caused approximately a 50% decrease in HCV cell-to-cell spread. Interestingly, this decrease in HCV cell-to-cell was stronger than the decrease detected after treatment with GI254023X (Fig 4B). As the reduction of ADAM10 surface expression upon BB-94 treatment was comparable to GI254023X (Fig 4C), this suggests that several matrix metalloproteinases could serve as HCV co-factors [65]. In hepatoma cells and primary hepatocytes, *ADAM10* and its closest relative *ADAM17* (also recognized as tumor necrosis factor α (TNFα) converting enzyme or *TACE*) show the highest expression levels (S1 Table). To assess a possible involvement of ADAM17, we silenced it in human hepatoma cells and assessed HCV infectivity. siRNA treatment mildly decreased ADAM17 surface expression levels (Fig 4E). In addition, HCV infectivity was slightly reduced in *ADAM17* silenced cells (Fig 4D), but not as pronounced as in *ADAM10* silenced cells, suggesting that ADAM10 is the main sheddase operating during HCV entry.

### ADAM10 promotes EGFR family member activation via E-cadherin and EGF ectodomain shedding in human hepatoma cells

As EGFR activation is required for productive HCV entry and ADAMs can shed EGF, we asked if ADAM10 also transactivates EGFR in human hepatoma cells. Similarly, ADAM10 sheds E-cadherin, which activates the EGFR family member ErbB2/3. Both EGFR family members induce a signaling cascade leading to phosphorylation of the ERK1/2 kinase (Fig 4F) [21]. To study a possible impairment of EGFR activation upon ADAM10 inhibition in hepatoma cells, we assessed phosphorylation of ERK1/2 by antibody staining and flow cytometry. As expected, EGFR stimulation with recombinant EGF leads to phosphorylation of ERK1/2 (Fig 4G). When treating Huh-7.5-Fluc cells with ADAM10 inhibitor with and without serum starvation, EGF-induced ERK1/2 phosphorylation decreased by 25% compared to the solvent control (Fig 4H). This observation was independent of serum starvation. We confirmed EGF dependent ERK1/2 phosphorylation upon EGF stimulation by immunoblot (Fig 4I and 4J). Next, we tested if ADAM10 inhibition would decrease EGFR activation measured by its autophosphorylation. EGFR autophosphorylation was not significantly reduced upon ADAM10 inhibition measured by antibody staining and flow cytometry (Fig 4K) and immunoblot (Fig 4L). This suggests that ADAM10 substrates other than EGF or a mere scaffolding function of ADAM10 mediated the observed enhancement of HCV infection.

To investigate if ADAM10 sheddase activity promoted HCV uptake and spread, we complemented *ADAM10* knockout cells with the three most prominent soluble substrates of ADAM10, tumor necrosis factor (TNF-α), EGF and E-cadherin. While TNF-α and EGF partially rescued the *ADAM10* knockout phenotype, E-cadherin fully restored HCV infection (Fig 4M). This effect was not due to increased cell proliferation upon growth factor stimulation as assessed by a metabolic activity assay (Fig 4N). Of note, we detected high variability between biological replicates in *ADAM10* knockout cells treated with TNF-α, EGF and E-cadherin, compared to unstimulated *ADAM10* knockout cells (Fig 4M). Collectively, these results suggest that ADAM10 promotes HCV infection via E-cadherin shedding and EGFR family member transactivation.

### ADAM10 is dispensable for infection with unrelated enveloped RNA viruses

**Fig 5:**
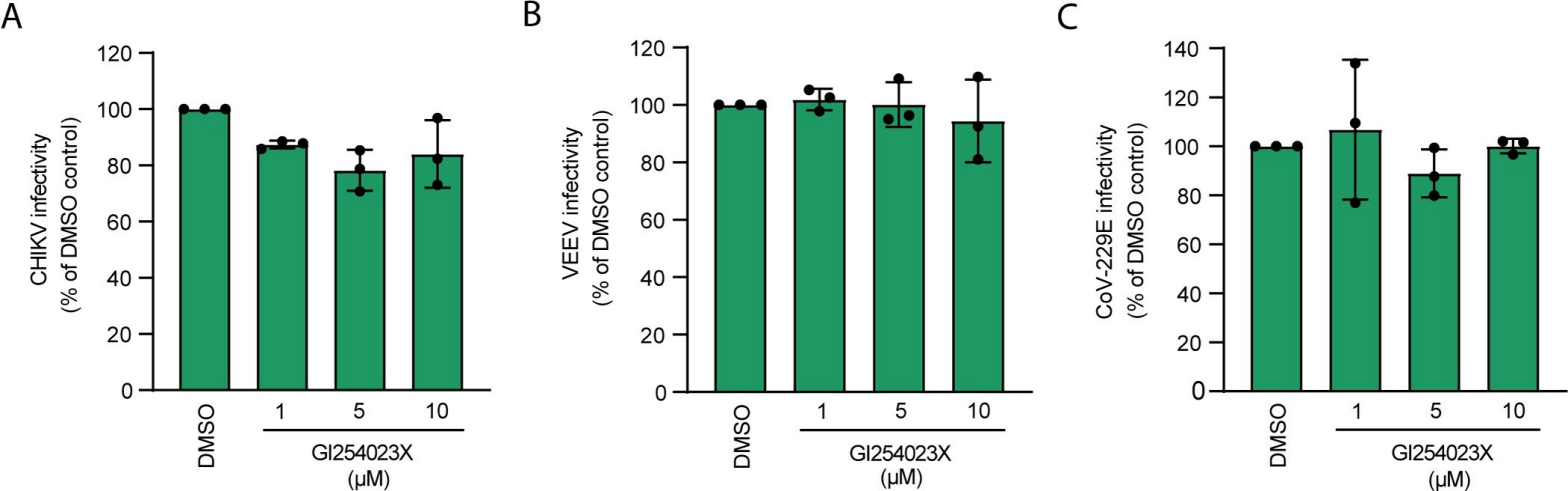
ADAM10 is dispensable for infection with alphaviruses and coronaviruses. **(A)** CHIKV-GFP (ECSA LR2006) infection of Huh-7.5-Fluc cells treated with the ADAM10 inhibitor GI254023X or DMSO (MOI 1). Infection levels at 24 h were measured by quantification of GFP signal and normalization to DMSO control. **(B)** VEEV-GFP (TC-83) infection (MOI 0.1) of Huh-7.5-Fluc cells treated with ADAM10 inhibitor or solvent control as in (A). **(C)** Renilla luciferase reporter hCoV-229E infection of Huh-7.5-Fluc cells treated as in (A). 72 h post infection, renilla luciferase activity was determined as a measure of infection. Data show the mean +/- SD of three biological replicates.

HCV belongs to the *Flaviviridae* family, which comprises enveloped positive-stranded RNA viruses. As EGFR family member activation is implicated in infection with viruses from various families including DNA and RNA viruses and ADAM17 was reported as a papilloma virus entry factor, we asked how specific the observed ADAM10 effect was. To that end, we tested enveloped positive sense RNA viruses from the *Togaviridae* and *Coronaviridae* families for their requirement for ADAM10. We treated Huh-7.5-Fluc cells with 1, 5 and 10 μM of the ADAM10 inhibitor GI254023X for 24 h and infected them with the *Togaviridae* family members chikungunya virus (CHIKV) and Venezuelan equine encephalitis virus (VEEV; vaccine strain TC-83) (Fig 5A and 5B) and the common cold *Coronaviridae* family member hCoV-229E (Fig 5C). ADAM10 inhibition neither affected susceptibility to CHIKV and VEEV, nor to hCoV-229E. Taken together, our data suggest that ADAM10 is an HCV specific host co-factor.

### ADAM10 facilitates infection of primary human hepatocytes

**Fig 6:**
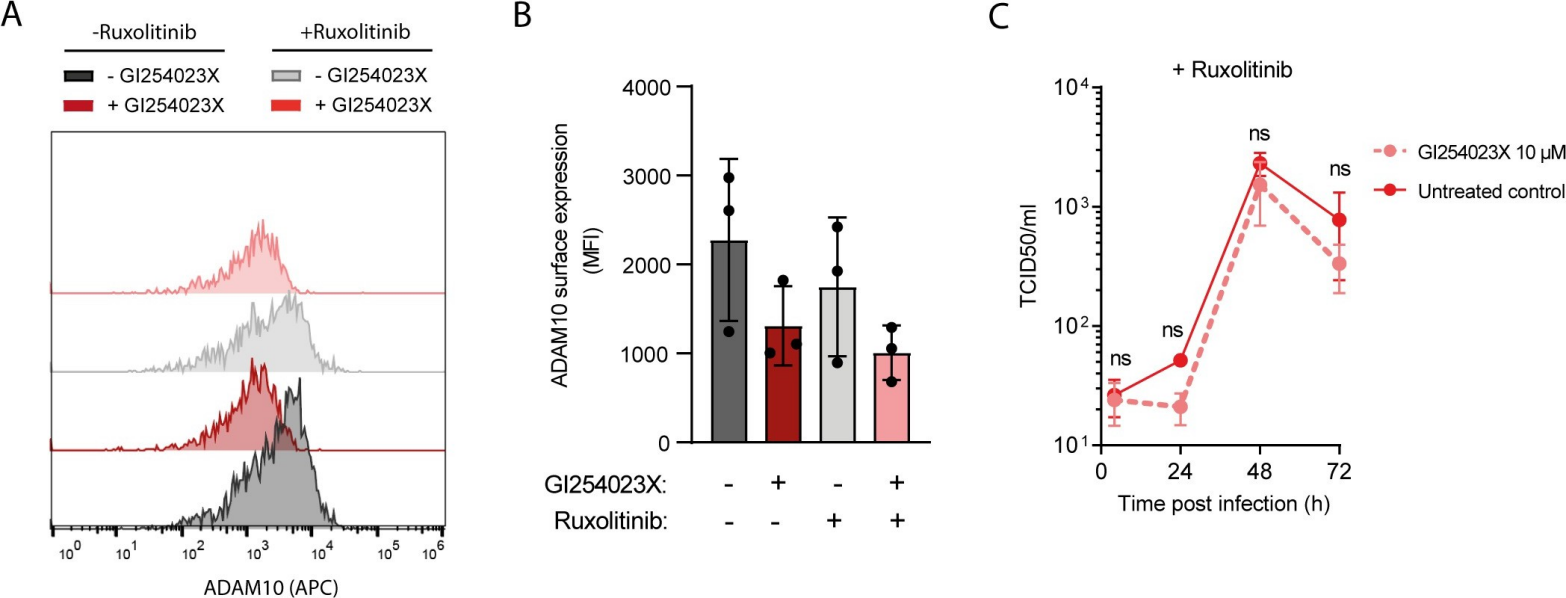
Effect of ADAM10 inhibition on HCV infection of primary human hepatocytes. **(A)** ADAM10 surface expression on primary human hepatocytes (PHH) upon ADAM10 inhibitor GI254023X treatment in presence or absence of JAK-STAT inhibitor ruxolitinib at 96 h post inhibitor treatment. **(B)** Quantification of ADAM10 surface staining as in (A) for PHH from three independent donors measured as mean fluorescence intensity (MFI). **(C)** Effect of ADAM10 inhibition on PHH infection with HCV upon pre-treatment with the Jak-STAT inhibitor ruxolitinib with and without ADAM10 inhibitor GI254023X for 24h. Cells were then infected with cell-culture adapted HCV P100 (supplemented with ruxolitinib and GI254023X as indicated) at MOI 1. Virus inoculum was removed 5h post infection and replaced with medium containing ruxolitinib and GI254023X as indicated. Virus supernatants were collected 24, 48 and 72 hpi and titrated on Huh-7.5 cells by TCID50. (C). Data show the mean +/- SEM of three biological replicates, i.e. PHH from three independent donors (B,C) or a representative experiment (A). Two-way ANOVA with Sidak´s multiple comparison test (ns = non-significant).

Animal models for HCV are limited due to the strong adaptation of the virus to humans. In order to elucidate the role of ADAM10 in HCV infection in a physiologically more relevant setting, we used PHH. To that end, we treated PHH from three independent donors with the JAK/STAT inhibitor Ruxolitinib to reduce the innate immune inhibition of HCV in presence or absence of the ADAM10 inhibitor GI254023X for 24 h. As expected, treatment with GI254023X decreased ADAM10 surface expression in PHH, independent of Ruxolitinib treatment (Fig 6A and 6B). Notably, the decrease in ADAM10 surface expression was not as pronounced as in Huh-7.5-FLuc cells with only 50% reduction in PHH as compared to 70% reduction in Huh-7.5-FLuc cells. When infecting PHH with the cell-culture adapted HCV P100pop, we observed lower HCV titers in the supernatants harvested from PHH treated with GI254023X at 4 and 72 hpi compared to the untreated control (Fig 6C) [66]. Decreased titers at early and late stages of infection point towards an effect on HCV entry and cell-to-cell spread as observed in Huh-7.5-Fluc cells. The effect seen in PHH is mild, possibly due to the lower efficiency of ADAM10 inhibition compared to the cell lines. Nonetheless, these findings are in line with our previous results obtained in Huh-7.5-Fluc cells and confirm that ADAM10 supports HCV cell entry and spread.

## Discussion

In this study, we identified ADAM10 as CD81 interaction partner and entry co-factor for HCV. Using pharmacological inhibition, silencing, and knockout technologies, we demonstrate a role of ADAM10 in HCV infection. By pharmacological time-of-addition experiments, we pinpoint the role of ADAM10 in HCV entry and cell-to-cell spread. Regarding the mechanism of ADAM10 mediated HCV host cell entry, we show that ADAM10 likely acts via E-cadherin shedding and EGFR family member activation, which is a critical step in HCV entry. Thus, this study could help clarify upstream mechanisms of EGFR family member activation, required for full susceptibility of cells to HCV.

HCV entry is a complex multistep process that requires four entry factors: SR-BI, CD81, claudin-1 and occludin. Previous studies have shown that these entry factors interact with each other and have additional interaction partners that contribute to susceptibility to HCV. For example, silencing the SR-BI partner PDZK1 impairs HCV entry [67]. Similarly, the interaction of claudin-1 with SEC24C is required for its transport to the plasma membrane and therefore for HCV entry [68]. In the case of CD81, four previously reported CD81 complex proteins, EGFR, SRFBP1, CAPN5 and CBLB, promote HCV entry [16,20]. While the role of EGFR in HCV entry is established, little information is available on mechanisms upstream of EGFR, which might impact susceptibility to HCV.

Here, we report ADAM10 as a novel CD81 protein complex component. In addition, we show that ADAM10 is broadly expressed in liver cells such as hepatocytes and Kupffer cells. Members of the ADAM family are membrane-anchored proteases that shed the ectodomain of other membrane-bound proteins that are receptor ligands. ADAMs are known host factors for other viruses; for example, ADAM17 mediates the assembly of the HPV entry platform, in a process that also involves EGFR [34]. ADAM17 also plays a role in the priming of the SARS-CoV-2 spike protein and is an attachment factor for classical swine fever virus [33] [69]. Regarding ADAM10, it is involved in replication of human immunodeficiency virus type-I [70] and required for syncytia formation (a consequence of cell-cell fusion) in lung cells during SARS-CoV-2 infection [33]. ADAM10 localization to adherens junctions of cell-cell contacts in addition to apical membranes is critical for this latter function [71]. Interestingly, the adherens junction protein E-cadherin is a reported entry factor for HCV [18] and is cleaved by ADAM10 [72]. In line with this, we find that ADAM10 is a host factor for HCV entry and cell-to-cell spread, two processes which take place at cellular junctions [73].

Mechanistically, ADAM10 and ADAM17 can shed EGFR family member ligands into the extracellular milieu leading to EGFR family member, i.e. EGFR and ErbB2/3, transactivation, which ultimately leads to ERK1/2 signaling [31]. EGFR is a known host factor for several viruses, which hijack its signaling pathway to enhance both viral entry and spread. Examples are Hepatitis B virus, which requires EGFR for internalization and vaccinia virus, which uses EGFR to enhance viral spread [74,75]. ADAM17 is the main sheddase of TGF-α and TNF-α, whereas ADAM10 primarily sheds EGF and E-cadherin [31,32,76]. In the case of ADAM17, its proteolytic activity triggers EGFR activation and is linked to its role as an HPV host factor [34]. In this study, we report that ADAM10 promotes HCV entry and spread. We show that ADAM10 inhibition reduces ERK1/2 pathway activation, a process previously shown to promote HCV entry [17,21]. However, as we detect this effect upon stimulation with a high concentration of EGF (1 μg/mL), we cannot exclude unspecific, EGFR independent effects. In line with this, we show that complementation with the ADAM10 substrate E-cadherin, but less so EGF, rescues the *ADAM10* knockout phenotype in HCV infection. In contrast, complementation with the ADAM17 substrates EGF and TNF-α partially rescues the *ADAM10* knockout phenotype in HCV infection. This latter effect should be further characterized to exclude effects due to variability among biological replicates. Clearly, E-cadherin, an EGFR family member ligand, can compensate *ADAM10* deficiency and promote HCV entry. Thus, our study provides a mechanistic upstream link to explain the role of EGFR family members in HCV entry.

ADAM10 activation is regulated by membrane asymmetry. Lipid scramblases, which lead to flipping of phosphatidylserine (PS) to the outer leaflet of the membrane, are thought to activate ADAM10 through a putative PS binding pocket in the ADAM10 stalk region [77]. Intriguingly, scramblases are reported HCV entry factors [78]. Thus, during HCV entry, scramblases may promote ADAM10 mediated EGFR family member transactivation. Notably, phospholipid scramblase 1 is an interferon-stimulated gene, which in turn promotes anti-HCV activity during replication [79]. Thus, lipid scrambling may fulfil antipodal roles during early and late steps of HCV liver cell infection. Our study demonstrates that ADAM10, most likely via E-cadherin shedding, promotes HCV entry into liver cells.

Here, we report that ADAM10 specifically promotes HCV entry but not entry of other enveloped RNA viruses. This is despite the fact that, components of the EGFR pathway like the epidermal growth factor receptor pathway substrate 15 (Eps 15) are involved in assembly of clathrin-coated vesicles, which constitute an entry mechanism hijacked by many RNA viruses including the here tested CHIKV, VEEV and common cold coronavirus 229E [80–83]. In line with a lack of evidence that EGFR family members are entry factors for CHIKV, VEEV or hCoV-229E, we show that ADAM10 inhibition does not decrease susceptibility to these viruses in hepatoma cells. However, this result should be challenged in other cell types, as VEEV is a neurotropic virus and hCoV-229E infects mainly human airway epithelia [84,85]. Future studies on these zoonotic RNA viruses will clarify a potential role of ADAM10 and ADAM17 in infection.

Our finding of ADAM10 as an HCV host co-factor mechanistically links several previously described entry co-factors including E-cadherin, EGFR and Eph2A [17]. ADAM10 can shed the ectodomain of E-cadherin [32], which is an HCV entry factor regulating the membrane localization of CLDN1, OCLN and Eph2A [18,86]. In our experimental system, ADAM10 did not affect CLDN1 or OCLN membrane expression. EphA2 is similar to EGFR a known co-factor for HCV entry [86]. Given the fact that EGFR is thought to co-internalize with HCV particles, our data indicate that E-cadherin shedding during HCV entry may facilitate EGFR and EphA2 internalization with the virus-receptor complex [87]. Clearly, this study is strengthening the role of ADAM family sheddases in regulating plasma membrane proteins during virus entry. The tight connection of tetraspanins and ADAMs has previously been described (reviewed in [88]). In particular, tetraspanins can regulate the localization of ADAMs and thereby target them to different substrates. CD81 is not implicated in changing the overall membrane localization of ADAM10. Nonetheless, our data collectively suggests that CD81 clusters HCV entry factors including EGFR family member receptors and ADAM10 thereby providing an entry platform for HCV.

Similar to EGFR antagonists, ADAM10 inhibitors are tested as anti-cancer drugs in randomized clinical trials [89]. Recently, EGFR inhibition showed promising results for preventing hepatocellular carcinoma in chronic HCV patients [73]. In this phase Ib prospective randomized double-blind placebo-controlled study the EGFR inhibitor erlotinib was safe and showed anti-HCV activity in non-cirrhotic patients. Whether progression to hepatocellular carcinoma could be reduced by erlotinib treatment is currently not known. Notably, E-cadherin is implicated in HCV driven epithelial to mesenchymal transition [18]. In the light of our results, ADAM10 inhibition may present a complementary strategy to block the ADAM10 –E-cadherin–EGFR family member axis and thereby not only execute anti-HCV activity but also reduce the risk of cancer development in chronic HCV patients. If in the era of highly effective direct acting antivirals against HCV such an alternative strategy is needed, remains to be seen.

HCV remains a major health threat, with chronic hepatitis being a leading cause of hepatocellular carcinoma [4]. The lack of a vaccine is still hampering global control of hepatitis C. Our study provides an example that additional pieces to the puzzle of the multifactorial HCV entry process can be discovered. Identification of these factors may help understand processes like HCV immune evasion via direct cell-to-cell spread and ultimately, promote development of orthogonal treatment options and a vaccine.

## Supporting information

S1 Fig Impact of GI254023X and BB-94 treatment on cell viability of Huh-7.5-Fluc cells. **(A)** Huh-7.5-Fluc cells were treated with 1, 5, 10, 15, 20, 25, 30, 40 and 50 μM of GI254023X vs DMSO for 20, 48, 72 and 96 h. At each time point, cell viability was measured using MTT assay. **(B)** Huh-7.5-Fluc cells were treated with 1, 5 and 10 μM of BB-94 for 72 h. 72 hours post treatment, cell viability was measured using MTT assay.
(TIF)

S2 Fig Treatment with low doses of GI254023X causes a mild decrease in ADAM10 surface expression that correlates with a marked impairment of HCV infectivity. **(A)** Huh-7.5-Fluc cells were treated with 100 nm, 500 nM or 10 μM GI254023X or DMSO for 72 h. ADAM10 surface expression was then measured by antibody staining and flow cytometry and shown as percentage of DMSO control. **(B)** Huh-7.5-Fluc cells were treated with GI254023X or DMSO as in (A). 16 h post treatment, cells were infected with JcR2a supplemented with 100 or 500 nM GI254023X or DMSO. ITX5061 was added as a positive control. HCV infection was quantified 72 h post infection as renilla luciferase activity. **(C)** Huh-7.5-Fluc cells were treated with GI254023X as in (B). 72 h post treatment, cell viability was assessed performing MTT assay. Cell viability values are shown as percentage of DMSO control. Data show the mean +/- SD of three biological replicates. One-way ANOVA with Dunnett´s multiple comparison test *** P < 0.001
(TIF)

S1 Table Transcript levels of ADAM family members in hepatoma cells and primary human hepatocytes (PHH). Reads per kilobase of transcript per million reads mapped (RPKM) showing transcript levels for several genes belonging to the ADAM family, highlighting in bold the data corresponding to ADAM10 and ADAM17. Higher expression values were detected for ADAM10 compared to ADAM17 [58].
(DOCX)
