# Supplementary figures and images for: The matrix metalloproteinase ADAM10 supports hepatitis C virus entry and cell-to-cell spread via its sheddase activity
Source: PLoS Pathog. 2023 Nov 15;19(11):e1011759. doi: 10.1371/journal.ppat.1011759 (PMC10650992; doi:10.1371/journal.ppat.1011759)

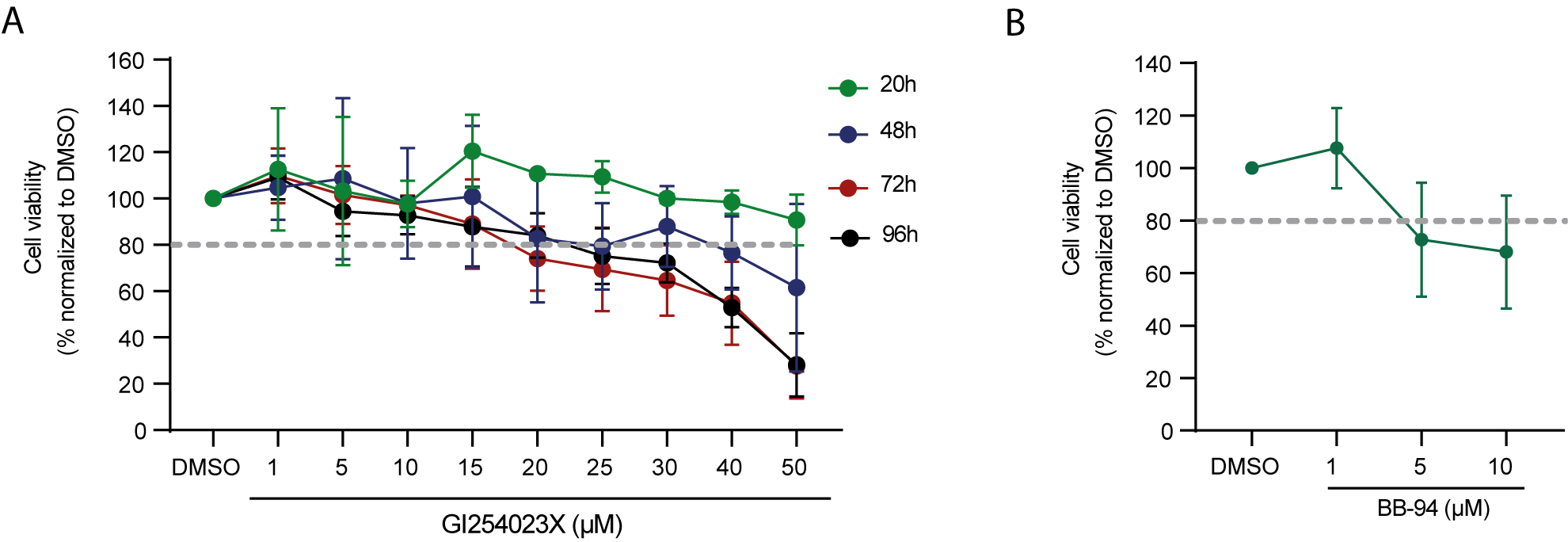

Supplement: S1 Fig — (A) Huh-7.5-Fluc cells were treated with 1, 5, 10, 15, 20, 25, 30, 40 and 50 μM of GI254023X vs DMSO for 20, 48, 72 and 96 h. At each time point, cell viability was measured using MTT assay. (B) Huh-7.5-Fluc cells were treated with 1, 5 and 10 μM of BB-94 for 72 h. 72 hours post treatment, cell viability was measured using MTT assay. (TIF) [file ppat.1011759.s001.tif]

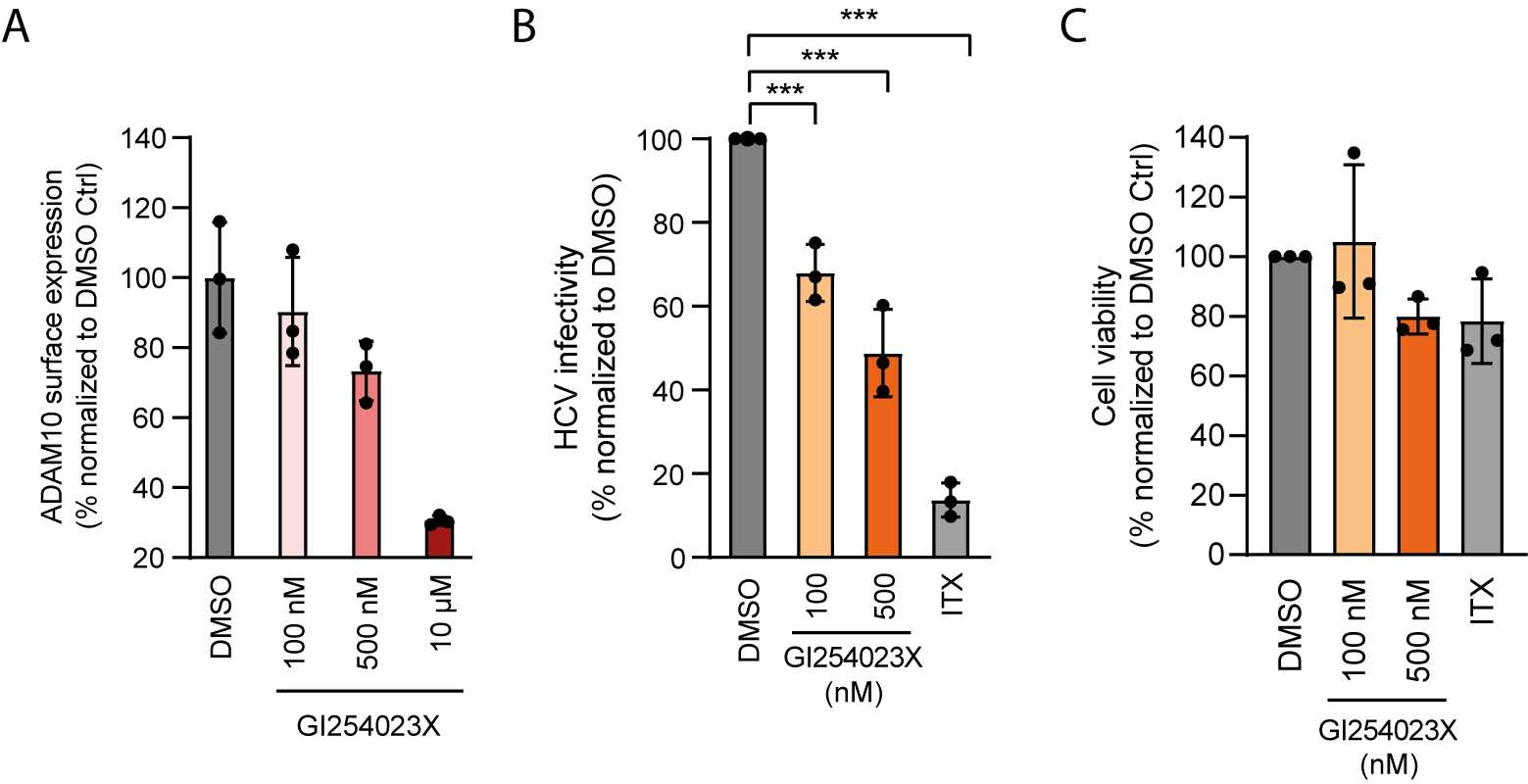

Supplement: S2 Fig — (A) Huh-7.5-Fluc cells were treated with 100 nm, 500 nM or 10 μM GI254023X or DMSO for 72 h. ADAM10 surface expression was then measured by antibody staining and flow cytometry and shown as percentage of DMSO control. (B) Huh-7.5-Fluc cells were treated with GI254023X or DMSO as in (A). 16 h post treatment, cells were infected with JcR2a supplemented with 100 or 500 nM GI254023X or DMSO. ITX5061 was added as a positive control. HCV infection was quantified 72 h post infection as renilla luciferase activity. (C) Huh-7.5-Fluc cells were treated with GI254023X as in (B). 72 h post treatment, cell viability was assessed performing MTT assay. Cell viability values are shown as percentage of DMSO control. Data show the mean +/- SD of three biological replicates. One-way ANOVA with Dunnett´s multiple comparison test *** P < 0.001 (TIF) [file ppat.1011759.s002.tif]
