# Supplementary material for: The matrix metalloproteinase ADAM10 supports hepatitis C virus entry and cell-to-cell spread via its sheddase activity
Source: PLoS Pathog. 2023 Nov 15;19(11):e1011759. doi: 10.1371/journal.ppat.1011759 (PMC10650992; doi:10.1371/journal.ppat.1011759)
Supplement: S1 Table — Reads per kilobase of transcript per million reads mapped (RPKM) showing transcript levels for several genes belonging to the ADAM family, highlighting in bold the data corresponding to ADAM10 and ADAM17. Higher expression values were detected for ADAM10 compared to ADAM17 [58]. (DOCX) [file ppat.1011759.s003.docx]

| *Gene* | Huh7.5 | PHH (donor 1) | PHH (donor 2) | PHH (donor 3) |
| --- | --- | --- | --- | --- |
| *ADAM10* | **9.319873** | **11.02125** | **6.138738** | **14.38087** |
| *ADAM11* | 0.082497 | 0.009002 | 0.004224 | 0 |
| *ADAM12* | 0 | 0.087102 | 0.016349 | 0.084175 |
| *ADAM15* | 0.764914 | 0.694496 | 1.040385 | 0.912828 |
| *ADAM17* | **4.977838** | **2.863541** | **3.084417** | **6.83253** |
| *ADAM18* | 0.006859 | 0 | 0 | 0 |
| *ADAM19* | 0.057844 | 1.73492 | 1.296278 | 2.224727 |
